# Multiparametric MR Imaging Features of Primary CNS Lymphomas

**DOI:** 10.3389/fsurg.2022.887249

**Published:** 2022-04-18

**Authors:** Rustam Talybov, Ozal Beylerli, Vadim Mochalov, Alexey Prokopenko, Tatiana Ilyasova, Tatiana Trofimova, Albert Sufianov, Yang Guang

**Affiliations:** ^1^Federal Center of Neurosurgery, Tyumen, Russia; ^2^Bashkir State Medical University, Ufa, Russia; ^3^V.M. Bekhterev Psychoneurological Research Institute, St. Petersburg, Russia; ^4^Department of Neurosurgery, Sechenov First Moscow State Medical University (Sechenov University), Moscow, Russia; ^5^Department of Neurosurgery, The First Affiliated Hospital of Harbin Medical University, Harbin, China; ^6^Institute of Brain Science, Harbin Medical University, Harbin, China

**Keywords:** CT, MRI, neuroradiology, perfusion, PCNSL

## Abstract

**Objective:**

Primary central nervous system lymphomas (PCNS) are relatively rare tumors, accounting for about 4% of all brain tumors. On neuroimaging, they are characterized by a low MR signal in T1, isointense in T2, bright uniform contrast enhancement, and diffusion restriction. The aim of this study is to note the lack of effectiveness of the MR/CT perfusion technique in complex multiparametric imaging in the differential diagnosis of primary lymphomas of the central nervous system in comparison with highly malignant gliomas and brain metastases.

**Materials and Methods:**

This prospective study included 80 patients with CNS tumors examined/operated at the Federal Center for Neurosurgery (Tyumen, Russia) from 2018 to 2021. The patients were divided into 4 groups: group 1 consisted of 33 cases with primary CNS lymphomas (10 cases with atypical manifestations according to perfusion parameters and 23 cases of classic CNS lymphomas), group 2 with anaplastic astrocytomas—14 cases, group 3—23 cases with glioblastomas and group 4—10 cases with solitary metastatic lesions. The study was carried out on a General Electric Discovery W750 3T magnetic resonance tomograph, a Canon Aquilion One multispiral X-ray computed tomograph (Gadovist 7.5 ml, Yomeron 400 mg−50 ml). Additionally, immunohistochemical analysis was carried out with the following markers: CD3, CD20, CD34, Ki-67, VEGF.

**Results:**

It has been established that MR/CT perfusion is not a highly sensitive method for visualizing primary CNS lymphomas, as previously thought, but at the same time, the method has a number of undeniable advantages that make it indispensable in the algorithm of a complex multiparametric diagnostic approach for this type of tumor. Nevertheless, PLCNS is characterized by an atypical manifestation, which is an exception to the rule.

**Conclusions:**

The possibilities of neuroimaging of primary lymphomas, even with the use of improved techniques for collecting MR/CT data, are limited and do not always allow reliable differentiation from other neoplasms.

## Introduction

Lymphomas of the central nervous system are divided into two subgroups: primary and secondary. Primary central nervous system lymphomas (PCNSL) are relatively rare, usually highly malignant tumors with an average lifespan of up to 30 months. Even less common are low-grade primary lymphomas, which have an indeterminate incidence. Secondary lymphomas are caused by the spread of a systemic lesion that originated outside the central nervous system, as part of its progression or as an isolated recurrence. The frequency of occurrence strictly depends on the histological subtype (1–4). There are difficulties in making a diagnosis of central nervous system lymphoma with a “standard” Magnetic resonance imaging (MRI) study (1, 2). Classical features with high specificity are found in immunocompetent patients who have not received therapy; in other cases, characteristic semiotics are absent (5) and are often similar to manifestations of highly malignant gliomas. At the same time, the differential diagnosis of these neoplasms is extremely important, since therapy and prognosis have significant differences (2, 6).

PCNSL is characterized by a classic group of signs due to histological features (hypercellularity, high nuclear/cytoplasmic ratio, disintegration of the blood-brain barrier) (7, 8). In immunocompetent patients, the lesion is usually solitary [multifocality occurs in 20–40% (5)] with intraaxial localization [dural lymphoma is a rare subtype (9)]. The changes are located in the periventricular white matter or in the superficial parts of the brain parenchyma (5, 8, 10), the lesion is mainly supratentorial, less often the stem and cerebellum are involved in the process, and rarely the spinal cord (11, 12). Quite typical spread to the contralateral side along the fibers of the corpus callosum, restriction of diffusion [low values on the Apparent diffusion coefficient (ADC) map, lower than malignant gliomas and metastases (8, 13–16)], mild perifocal edema (5, 17), intense and homogeneous accumulation of contrast agent (5, 8). Sometimes there is a pattern of annular contrast (8, 11) or linear contrast enhancement along the perivascular spaces (10, 18). Hemorrhages are rarely visualized (5, 19). Multifocal lesions are more typical for human immunodeficiency virus (HIV)-associated lymphoma (30–80%), often with uneven, peripheral or annular contrast enhancement due to necrotic changes (20–22). Spontaneous hemorrhages are also more frequently observed in immunocompromised patients (20). Secondary lymphoma is predominantly characterized by leptomeningeal spread in about two-thirds of cases, while intraaxial involvement occurs in only 30% of cases (23). Leptomeningeal metastasis often involves the cisternal portions of the cranial nerves, spinal cord sheaths, and spinal roots (24), which is no different from leptomeningeal processes of any other etiology.

In the context of the correlation between the degree of biological aggression of the tumor and its angiogenic activity, perfusion studies provide valuable information on the delivery of arterial blood to the capillary bed of the tumor tissue, including such parameters as cerebral blood volume (CBV) and cerebral blood flow (CBF) (8, 25–27). Primary lymphomas show low maximum relative CBV values (1, 8, 28–30), which is consistent with histopathological findings indicating the absence of neovascularization (1, 6, 8, 28–30), and is fundamentally different from malignant gliomas or solitary metastases. This finding explains the hypothesis of a lower microvascular density of lymphoma, since neovascularization is not a histological characteristic of PCNSL. In addition, low values of the percentage of signal intensity recovery are noted, also presumably due to massive leakage of the contrast agent into the interstitial space during the passage of the bolus through highly permeable vessels. The latter is explained by the angiocentric nature of growth, in which tumor cells are grouped around already existing brain vessels, inducing their immunoreactive changes (1, 2, 14). There is a known correlation of parameters measured during computed tomography (CT)/MR perfusion with the mitotic index and the degree of malignancy of gliomas. Thus, it has been established that tumor CBV normalized to CBV of the unaltered white matter of the contralateral hemisphere is a biomarker of glioma neoangiogenesis (31). It is believed that improved imaging techniques implemented in multiparametric mapping (mpMRI) can reliably distinguish central nervous system lymphoma from high-grade glioma and metastases (8, 30, 32), but the relationship between perfusion parameters and lymphoma aggression has not been adequately studied.

## Materials and Methods

The study was approved by the local ethics committee of the Federal Center for Neurosurgery, Tyumen, Russia. Written informed consent for diagnostic manipulations was obtained from all participating patients. The total number of patients with PCNSL, gliomas (Grade 3–4) and MTS who received neurosurgical care in the Department of Neurooncology of the Federal Center for Neurosurgery, Tyumen, Russia. and a pathomorphological conclusion was 80 people (of which 51 were men, 29 were women), their age ranged from 32 to 82 years, mean age was 54 years, median was 58 years. Of these, the diagnosis of PLCNS was made in 33 patients, among whom 10 cases of lymphoma showed atypical signs according to perfusion. Anaplastic astrocytoma (AA) was diagnosed in 14 patients (not otherwise specified (NOS), isocitrate dehydrogenase (IDH) status was not determined), glioblastoma (GB)—in 23 patients (NOS, IDH status was not determined), solitary metastatic lesions (sMTS)—in 10 patients.

Patients with atypical manifestations of PCNSL perfusion made up a group of observations: a total of 10 people, aged from 32 to 71 years. Neurological manifestations differed depending on the localization of the tumor, a combination of motor and sensory disorders, indicating a multifocal lesion. Information on hormone therapy and HIV status was not available.

The results of MRI with contrast and dynamic dynamic susceptibility contrast (DSC-T2^*^) perfusion, multislice computed tomography (MSCT) with perfusion study were of decisive importance for the diagnosis. MRI was performed on a GE 3T Discovery W750 tomograph using an 8-channel head coil, MSCT was performed on a Canon Aqullion One machine, 640 slices. As a contrast agent in the MR study, the Gadovist paramagnetic was used with a dose calculation of 0.1 ml/kg IV; in the perfusion CT study, Iomeron 400 was used at a dose of 50 ml IV, injected into the cubital vein using an automatic injector with injection rate of 5 ml/s. The perfusion protocol included a dynamic series of CT sections performed every second for 60 s, performed by an interval type of scanning at an X-ray tube voltage of 80 kV and a current-time product of 150 mAc. The total radiation exposure (effective dose) for the entire CT examination was no more than 3–4 mSv. The MR study protocol included the following pulse sequences: gradient echo (T1-weighted images “BRAVO”), spin echo (T2-weighted images), susceptibility-weighted angiography (SWAN), Diffusion-weighted magnetic resonance imaging (DWI) with the construction of maps of the apparent diffusion coefficient (ADC), DSC-T2^*^. Image post-processing was carried out on GE “Advantage Window 4.5” and “Vitrea Advanced Visualization” graphics stations. Blood flow parameters were assessed using two perfusion maps: CBF—ml/100 g/min; CBV—ml/100 g. To normalize blood flow parameters, region of interest (ROI) was used in the intact white matter of the semioval centers (the value of blood flow in the tumor/value in the intact white matter). The normalized blood flow parameters were calculated as the ratio of the values of the parameters in the area of interest to the intact brain substance. Statistical processing of the obtained results was carried out using the methods of descriptive statistics and correlation analysis.

Eight patients underwent biopsy, of which in six cases the lesion was solitary and in two cases it was multifocal. Stereotactic biopsy was performed using 3D navigation (Brainlab). Verification of the obtained data was carried out using histological and immunohistochemical research methods. Immunohistochemical analysis included the following range of markers: CD3, CD20, CD34, Ki-67, vascular endothelial growth factor (VEGF).

Taking into account the similar imaging characteristics of PCNS lymphomas with high-grade gliomas (Grade 3–4) and solitary metastatic lesions, a detailed comparative analysis of all tumors was performed using both routine and specialized MR sequences: DWI, SWI or SWAN, dynamic contrasting with the construction of perfusion maps, which form the basis of complex mpMRI.

## Results

In the course of the study, an analysis of the MRI and CT perfusion data of 80 patients was performed, followed by the allocation of 4 groups based on the histological diagnosis: group 1—14 cases with AA, group 2—23 cases with GB, group 3—10 cases with (sMTS) and group 4—33 cases of PLCNS, divided into 2 subgroups: (a) classic—23 cases; (b) atypical—10 cases.

Parameters in group 1: blood flow velocity (BF) in anaplastic astrocytomas ranged from 32.2 to 190.8 ml/100 g/min, blood flow volume (BV)—from 0.53 to 4.79 ml/100 g. The averaged maximum and normalized blood flow values are shown in Table 1.

**Table 1:** Average absolute (BF, BV) and normalized (BFn, BVn) numerical values of blood flow parameters in tumors depending on histological affiliation.

**Type of tumor**	**BF ±StDev ml/100g/min**	**BFn ±StDev**	**BV ±StDev ml/100g/min**	**BVn ±StDev**
Anaplastic astrocytoma	156.25 ± 26.51	3.85 ± 0.62	20.64 ± 4.02	4.5 ± 1.01
Glioblastoma	290.78 ± 16.13	8.69 ± 1.02	35.61 ± 2.98	8.39 ± 0.73
Metastasis	300.12 ± 25.51	6.75 ± 0.67	37.62 ± 3.97	7.12 ± 1.04
Typical PCNSL	43.11 ± 3.84	1.05 ± 0.05	2.67 ± 0.19	0.92 ± 0.12
Atypical PCNSL	155.7 ± 18.03	4.7 ± 0.45	17.8 ± 1.51	5.9 ± 0.41

*StDev, statistical deviation, PCNSL, primary central nervous system lymphoma*.

The 2nd group of patients (with GB) was characterized by high absolute and normalized blood flow parameters.

In the 3rd group of patients (with solitary MTS), high rates of CBF and CBV were revealed.

The 4th group of patients (with PLCNS) showed both low (most of them—23 cases) and high (10 atypical cases) values on perfusion maps (Table 1).

**Figure 1:**
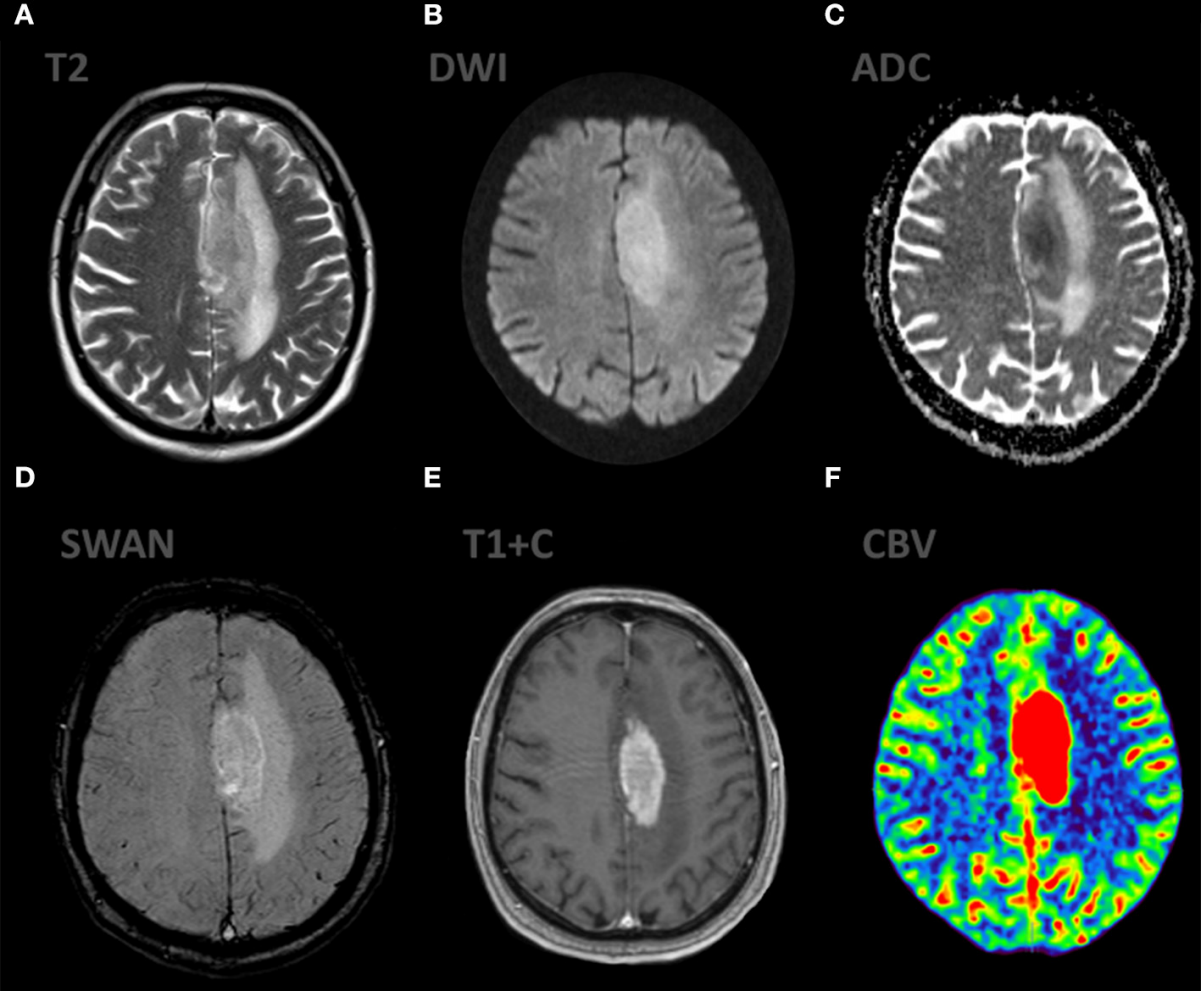
Patient V., male 61 y.o. presented with acute right sided weakness. Axial Brain MRI and CT-perfusion. MRI ax: **(A)** T2WI; 6, **(B)** DWI, **(C)** ADC, **(D)** SWAN, **(E)** T1 + CE. CT ax: **(F)** CT-perfusion (CBV map). There is a well circumscribed mass in the left frontal region with surrounding vasogenic edema on T2WI. The mass has low SI on ADC map indicating high cellular density. The contrast enhancement is bright and uniform. CT-perfusion map shows increased rCBV level.

**Figure 2:**
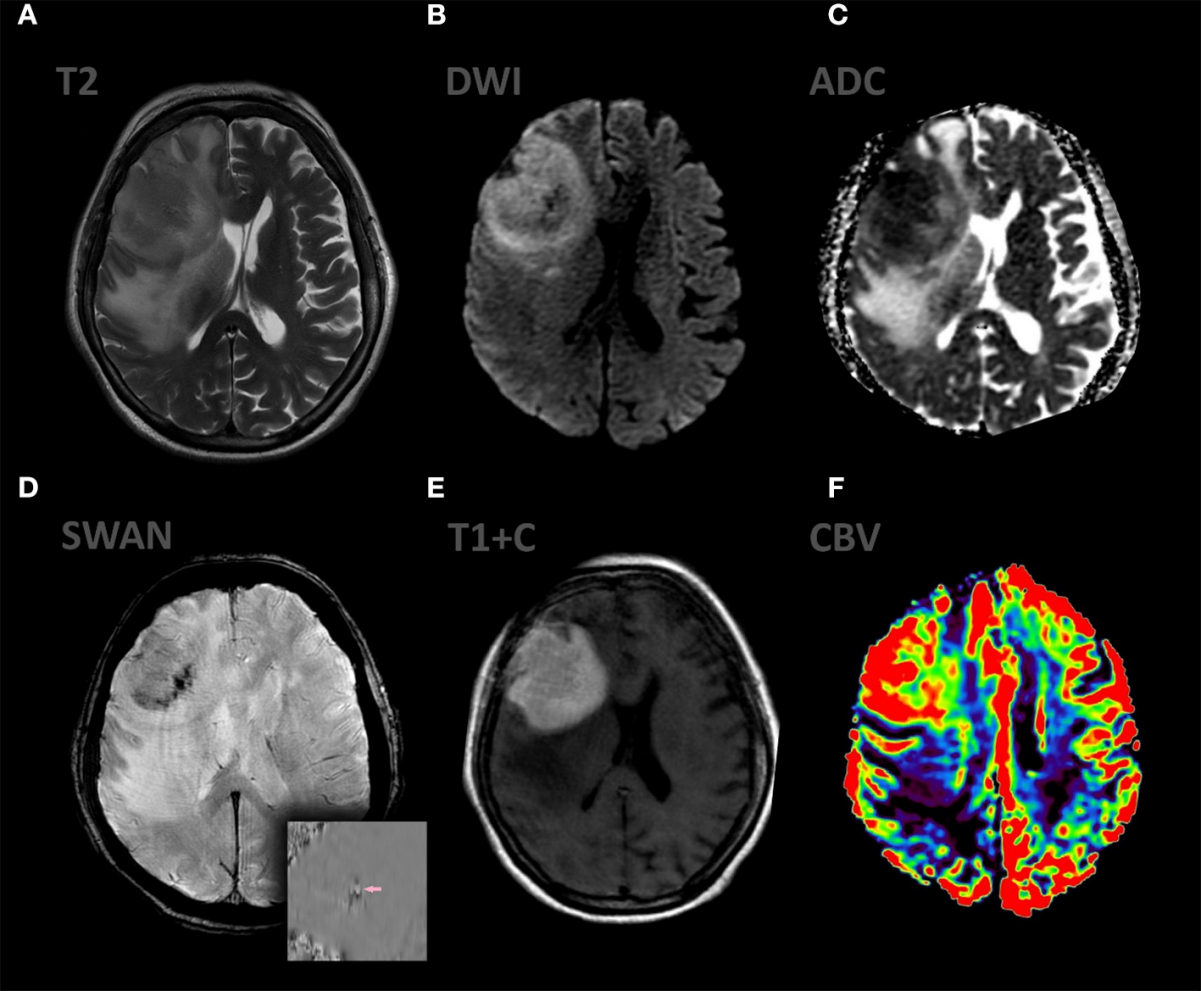
Patient A., female 67 y.o. presented with left sided weakness and altered mental status. MRI ax: **(A)** T2WI; 6, **(B)** DWI, **(C)** ADC, **(D)** SWAN, **(E)** T1 + C, **(F)** DSC-PWI. Axial Brain MRI: there is a large intraaxial mass in right frontal lobe with extensive peritumoral edema on T2WI and mild mass effect. The mass has restricted diffusion and bright, homogeneous contrast enhancement. Most of the tumor exhibits hyperperfusion. SWAN indicates small amounts of hemorrhage.

**Figure 3:**
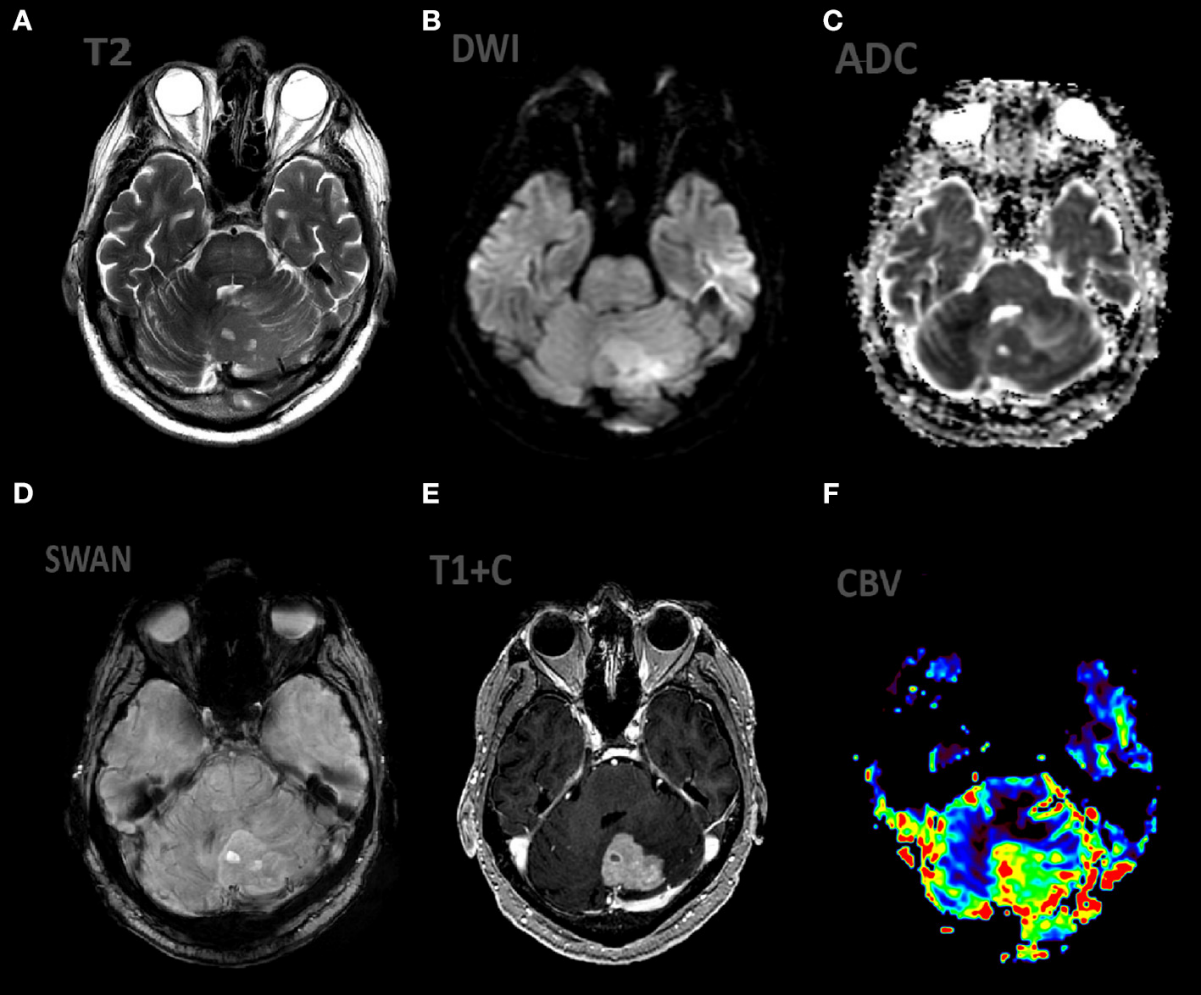
Patient V., male, 66 y.o. presented with gate disturbances. MRI ax: **(A)** T2WI; 6, **(B)** DWI, **(C)** ADC, **(D)** SWAN, **(E)** T1 + C, **(F)** DSC-PWI. Axial Brain MRI: there is lesion in periventricular white matter, the lesion involve left cerebellar peduncle, hemisphere. The lesion demonstrates high SI on T2WI, restricted diffusion (with low ADC values), homogeneous contrast enhancement, elevated rCBV values.

**Figure 4:**
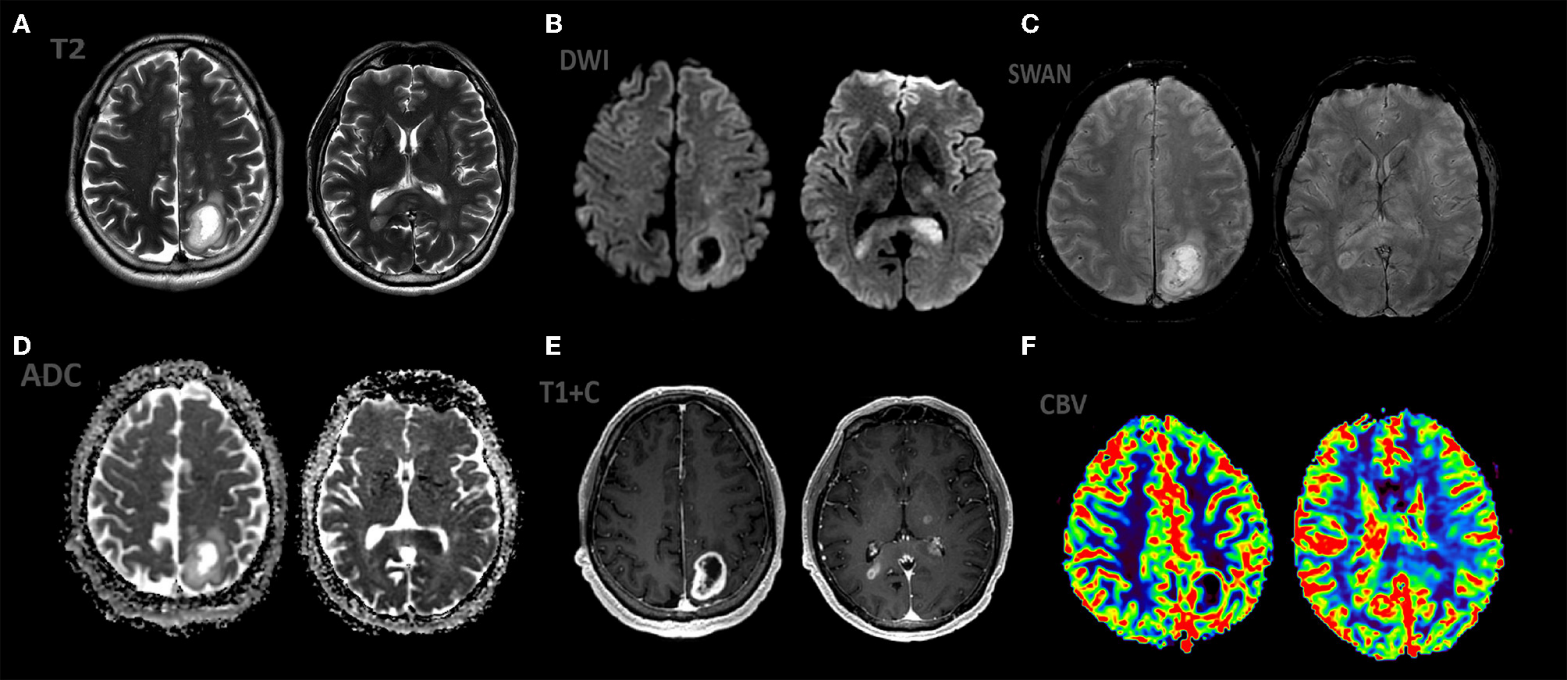
Patient S., male 55 y.o. presented with right sided weakness and memory loss. MRI ax: **(A)** T2WI; 6, **(B)** DWI, **(C)** ADC, **(D)** SWAN, **(E)** T1 + C, **(F)** DSC-PWI. Axial Brain MRI: there is a intraaxial mass in left parietal lobe with low peritumoral edema on T2WI, another mass in the splenium of the corpus callosum and left thalamus. All mass parts have restricted diffusion and bright, homogeneous contrast enhancement and hight rCBV values.

The results are presented in Table 2 and Figures 1–4.

**Table 2:** Key characteristics of observations of patients with primary cerebral lymphomas.

**Age**	**Male**	**Number of affected nodes**	**Localization (subcortical/periventricular)**	**ADC values x10-6 mm2/s**	**Severity of edema**	**Character contrasting**	**The presence of hemorrhages**	**Histological diagnosis**	**rCBV score vs. normal contralateral white matter (number of times)**
32	F	Multiple	Subcortical	450	Pronounced	Homogeneous	No	B-cell lymphoma	×6.0
47	M	Multiple	Subcortical	420	Pronounced	Homogeneous	Yes	B-cell lymphoma	×6.5
53	F	Multiple	Subcortical	457	Moderate	Homogeneous	No	Verification was not carried out	×7.5
55	M	Multiple	Subcortical Periventricular	500	Weak	Homogeneous	No	B-cell lymphoma	×5.0
59	M	Solitary	Periventricular	520	Weak	Homogeneous	No	Verification was not carried out	×5.1
61	F	solitary	periventricular	580	pronounced	homogeneous	No	B-cell lymphoma	×6.2
66	M	Solitary	Subcortical	570	Weak	Homogeneous	No	B-cell lymphoma	×7.0
67	F	Solitary	Subcortical	490	Pronounced	Homogeneous	Yes	B-cell lymphoma	×7.0
67	F	Solitary	Periventricular	500	Moderate	Homogeneous	Yes	B-cell lymphoma	×7.0
71	M	Solitary	Subcortical	539	Pronounced	Homogeneous	No	B-cell lymphoma	×3.0

*M, male; F, female; ADC, apparent diffusion coefficient; rCBV, relative cerebral blood volume*.

In the entire group of patients with suspected primary lymphoma, information about HIV status and hormonal therapy was missing. In six cases, the lesion was represented by a solitary tumor node with severe perifocal edema. In four cases, two or more tumor nodes were visualized against the background of edema. In all cases, the changes were characterized by intraaxial localization (periventricular and subcortical), intense and homogeneous contrast enhancement, and low ADC values. In three cases, hemorrhages were noted. MR DSC and CT perfusion showed hypervascularization with rCBV values ranging from 3.0 to 7.5 times compared to normal white matter.

**Figure 5:**
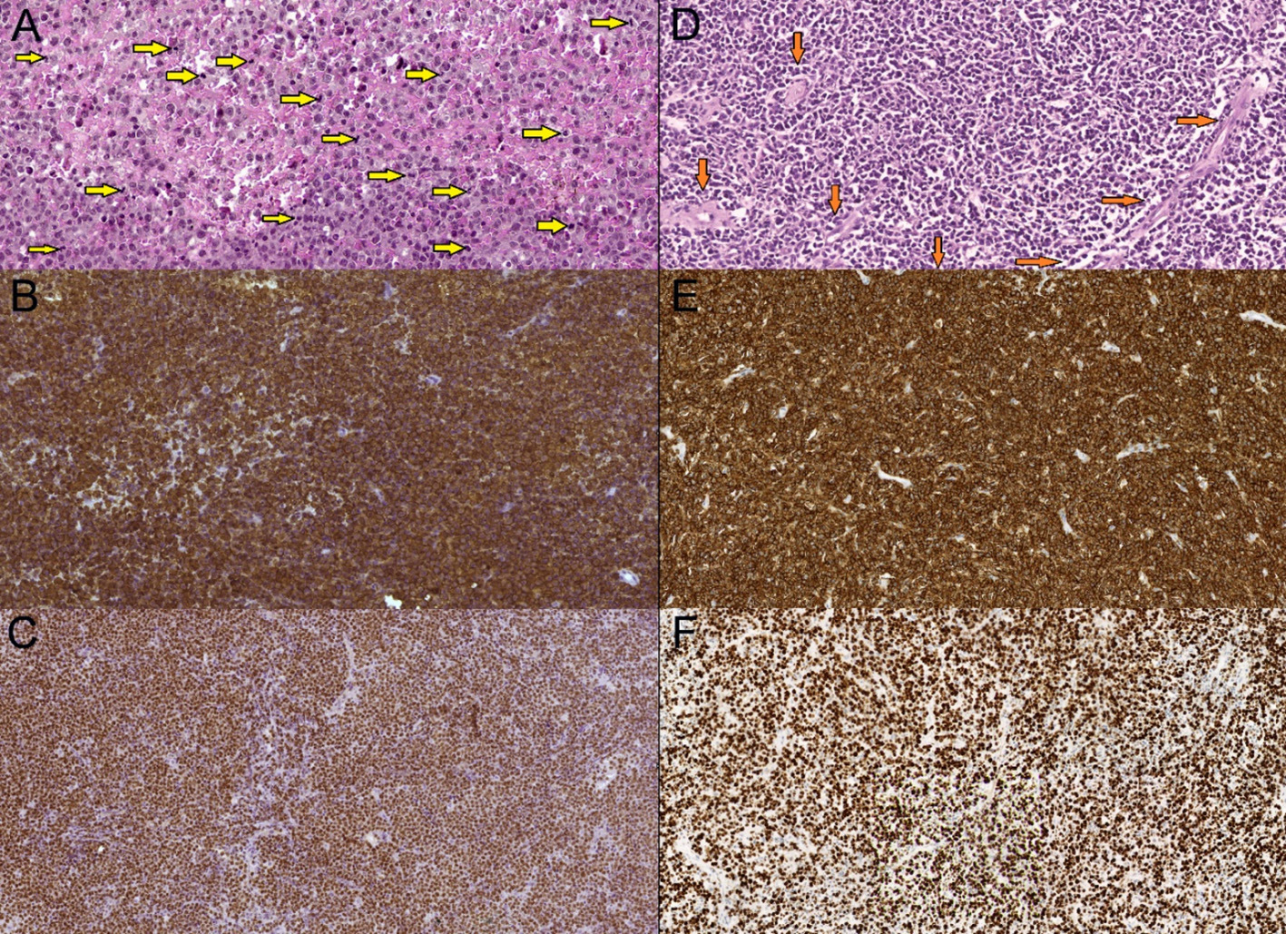
Primary diffuse large cell B-cell CNS lymphomas, mag. x20. **(A,D)**—hematoxilin-eosin stain—hypercellular tumorous tissue with infiltrative growth, cells have centroblasts morphology. Multiple mitoses present (A—yellow arrows), tumorous vessels (D—orange arrows). **(B,E)**—CD20 marker (B-lymphocytic antigen)—prominent membranes CD20+ expression. **(C,F)**—Ki-67 (MIB-1) marker—high proliferation index Ki-67—more than 90%.

**Figure 6:**
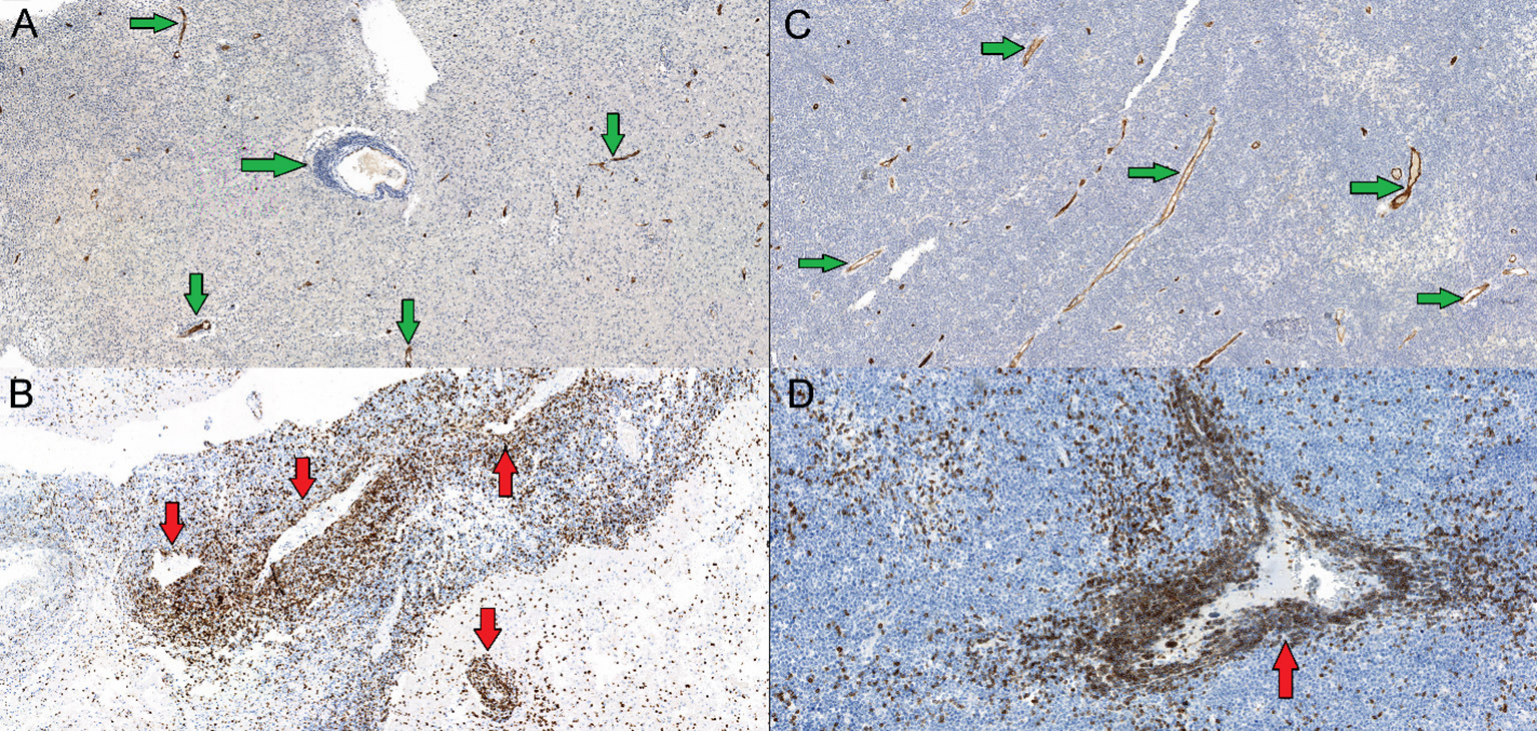
**(A,C)**—CD34 marker (hematopoetic and endothelial cells progenitor marker)—tumor is vascularised by low caliber capillaries and arterioles, endothelium expresses CD34+ (green arrows), proliferation is scarce. **(B,D)**—CD3 marker (T-cells co-receptor)—more wide vessels locally seen with an impression of an “empty lumen”, and abundant perivascularCD3+ T-lymphocytes infiltration caused by vessels walls increased permeability (red allows).

**Figure 7:**
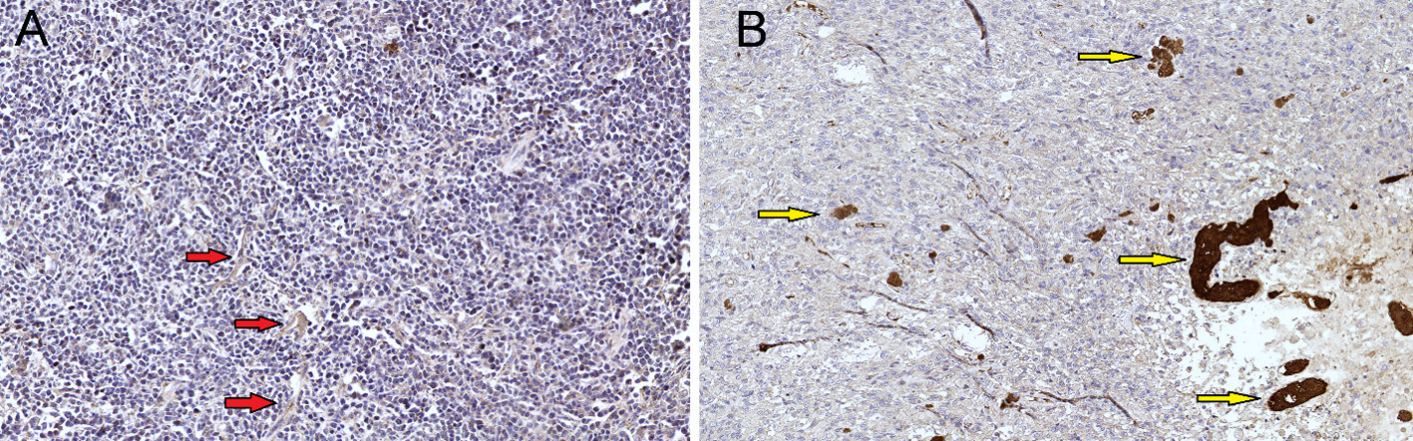
**(A)** Primary diffuse large cell B-cell CNS lymphomas, mag. × 20. VEGF marker, lymphomas showed no increased expression of VEGF (vascular endothelial growth factor) (red arrows). **(B)** GBM demonstrated expression of VEGF factor (control group) and showed massive antibody expression in proliferating vessels (yellow arrow).

Histological studies in all these cases revealed hypercellular tumor tissue with infiltrative growth, high mitotic activity and severe perivascular lymphocytic infiltration, increased endothelial permeability. In immunohistological studies, the tumor tissue totally expressed CD20+, the vascular endothelium was brightly stained in reaction to CD34+ and expressed extremely weakly VEGF. T-lymphocytes were positive in reaction with anti-CD3+. The proliferative activity index for Ki-67 was extremely high, over 90%. According to the results of immunohistological studies, lymphoproliferative diseases (primary B-cell lymphoma) were diagnosed (Figures 5–7).

## Discussion

**Figure 8:**
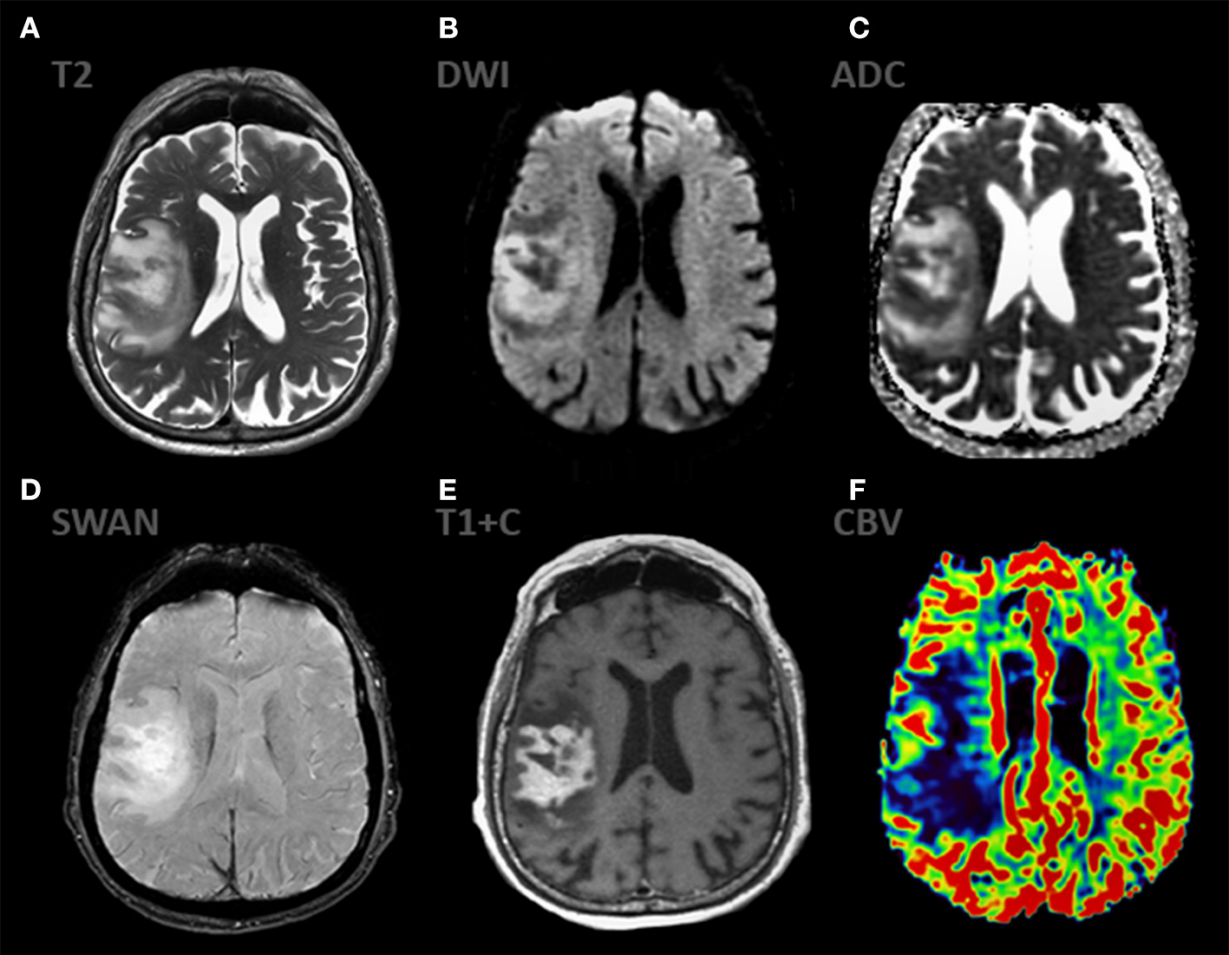
mpMRI ax: **(A)** T2WI; 6, **(B)** DWI, **(C)** ADC, **(D)** SWAN, **(E)** T1 + C, **(F)** DSC-PWI show classic appereance of PCNSL. The mass shows predominantly solid, intense contrast enhancement and has extensive peripheral T2-hyperintensiry. The enhancing component of the lesion demonstrates striking restricted diffusion (ADC 450-490 × 10^−6^ mm^2^/s) indicating high cellularity. The mass demonstrates low rCBV values.

Undoubtedly, modern qualitative and quantitative imaging tools implemented on CT and MRI cannot compete with invasive techniques in the reliability of determining the histopathological nature of the tumor. Despite this, the main non-invasive method for diagnosing brain tumors is MRI with complex mpMRI mapping (Figure 8). The classic imaging manifestations of primary lymphoma of the central nervous system include a group of rather specific criteria according to mpMRI mapping and are as follows: a mass showing a homogeneous and pronounced contrast enhancement, accompanied by moderate perifocal edema, usually without a mass effect, limiting the diffusion of molecules water with low ADC values within 400–600 × 10–6 mm^2^/s (0.4–0.6/100mm^3^), which has low values of rCBV (1, 26, 29–32). It is generally accepted that, when quantified based on CT and MR perfusion techniques, primary lymphomas show low maximum relative values of rCBV compared with poorly differentiated glial lesions and metastases (1, 8, 28–30, 32).

**Figure 9:**
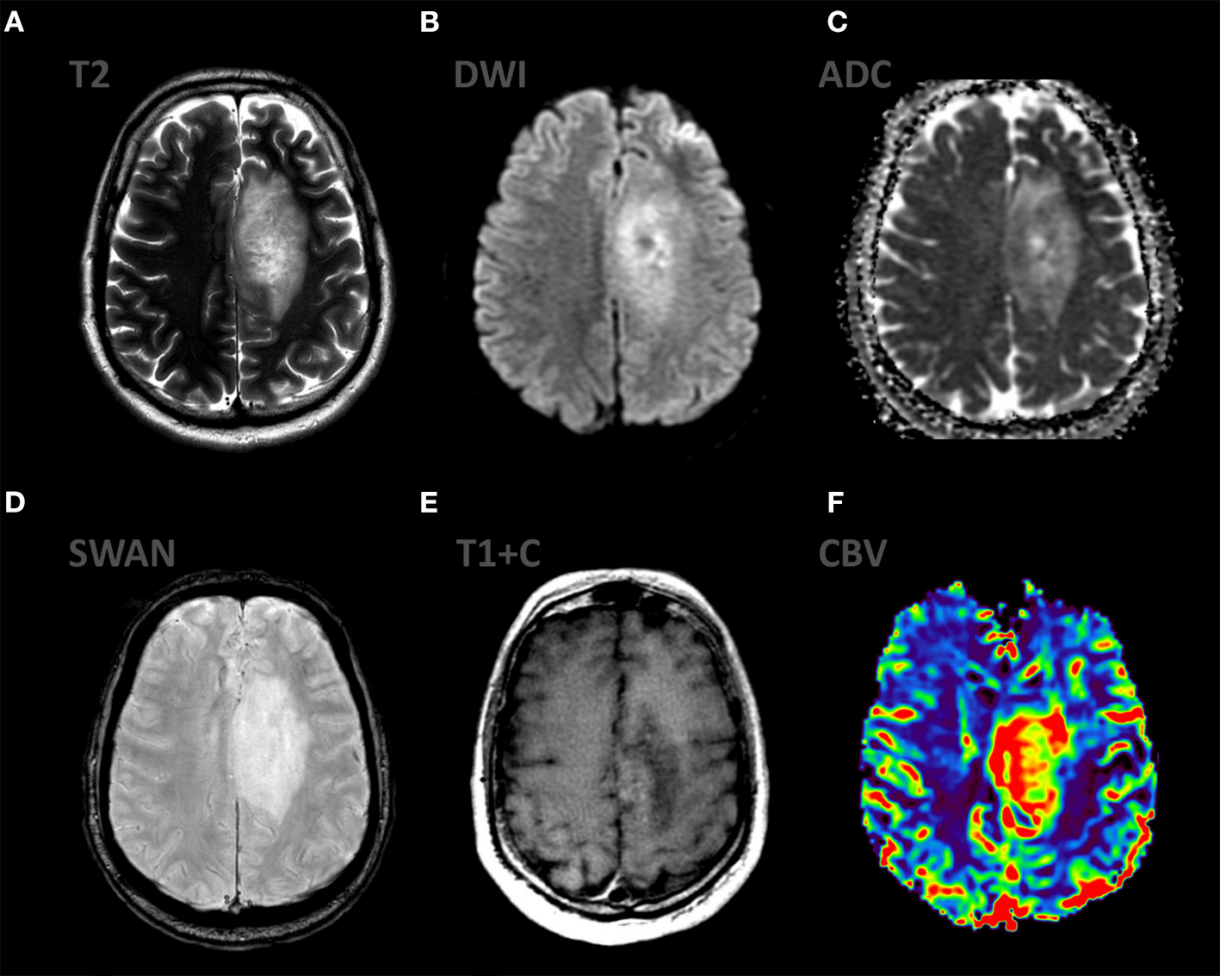
mpMRI ax: **(A)** T2WI; 6, **(B)** DWI, **(C)** ADC, **(D)** SWAN, **(E)** T1 + C, **(F)** DSC-PWI demonstrate the typical appereance of anaplastic astrocytoma (Grade III WHO 2016). There is infiltrative mass in the left frontal region with mild vasogenic edema on T2WI. This mass has low signal on ADC, partial enhancement and high rCBV values.

**Figure 10:**
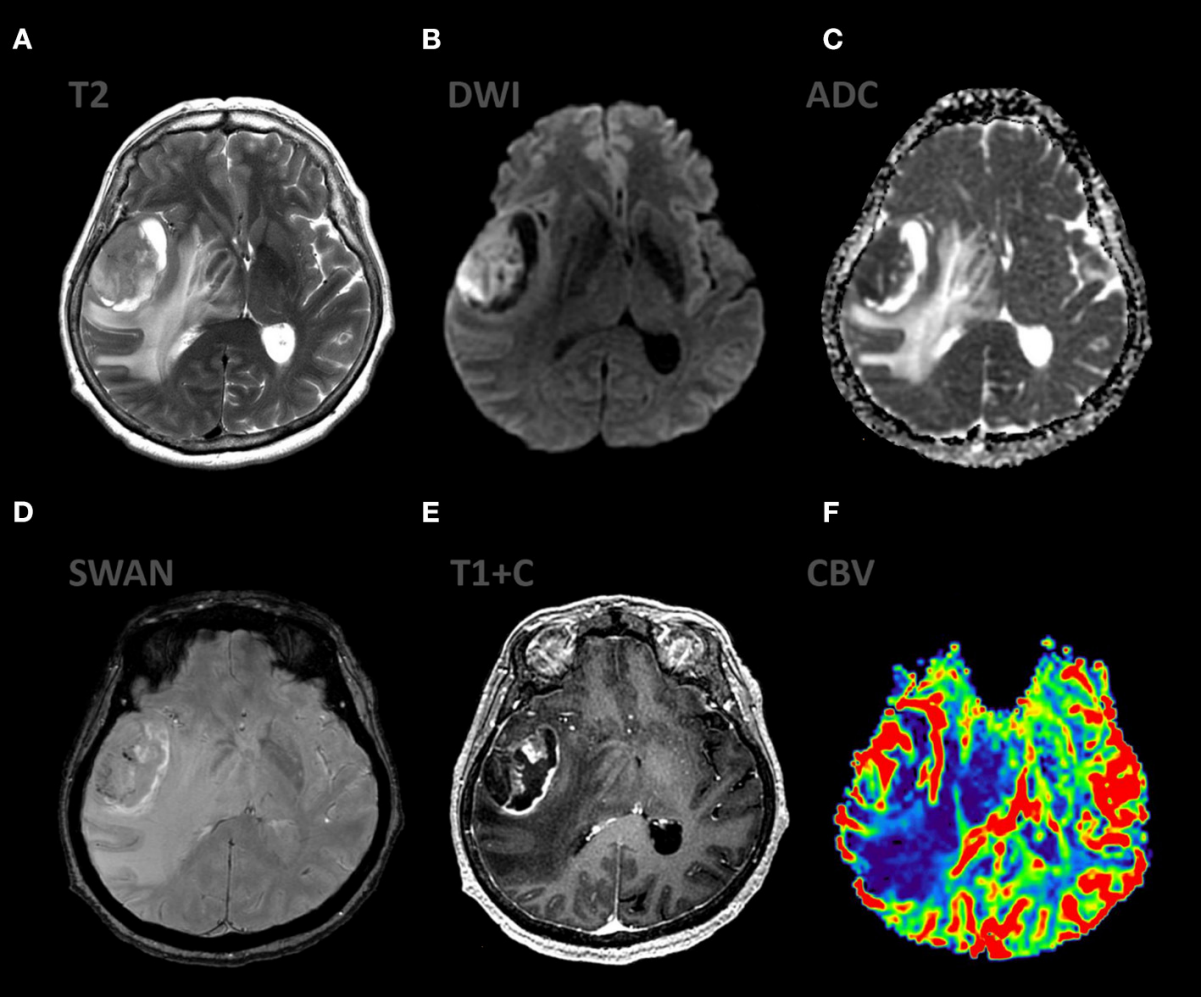
mpMRI ax: **(A)** T2WI; 6, **(B)** DWI, **(C)** ADC, **(D)** SWAN, **(E)** T1 + C, **(F)** DSC-PWI show the classic appereance of GBM (grade IV WHO 2016). The mass shows ring enhancing pattern associated with extensive peripheral T2-hyperintensity and moderate mass effect. The enhancing component of the lesion demonstrates restricted diffusion (ADC 800–900 × 10^−6^ mm^2^/s), also the mass shows high values rCBV.

The differential diagnostic range includes high grade gliomas (anaplastic astrocytoma and glioblastoma) and solitary metastatic lesions (Figures 9,10).

**Figure 11:**
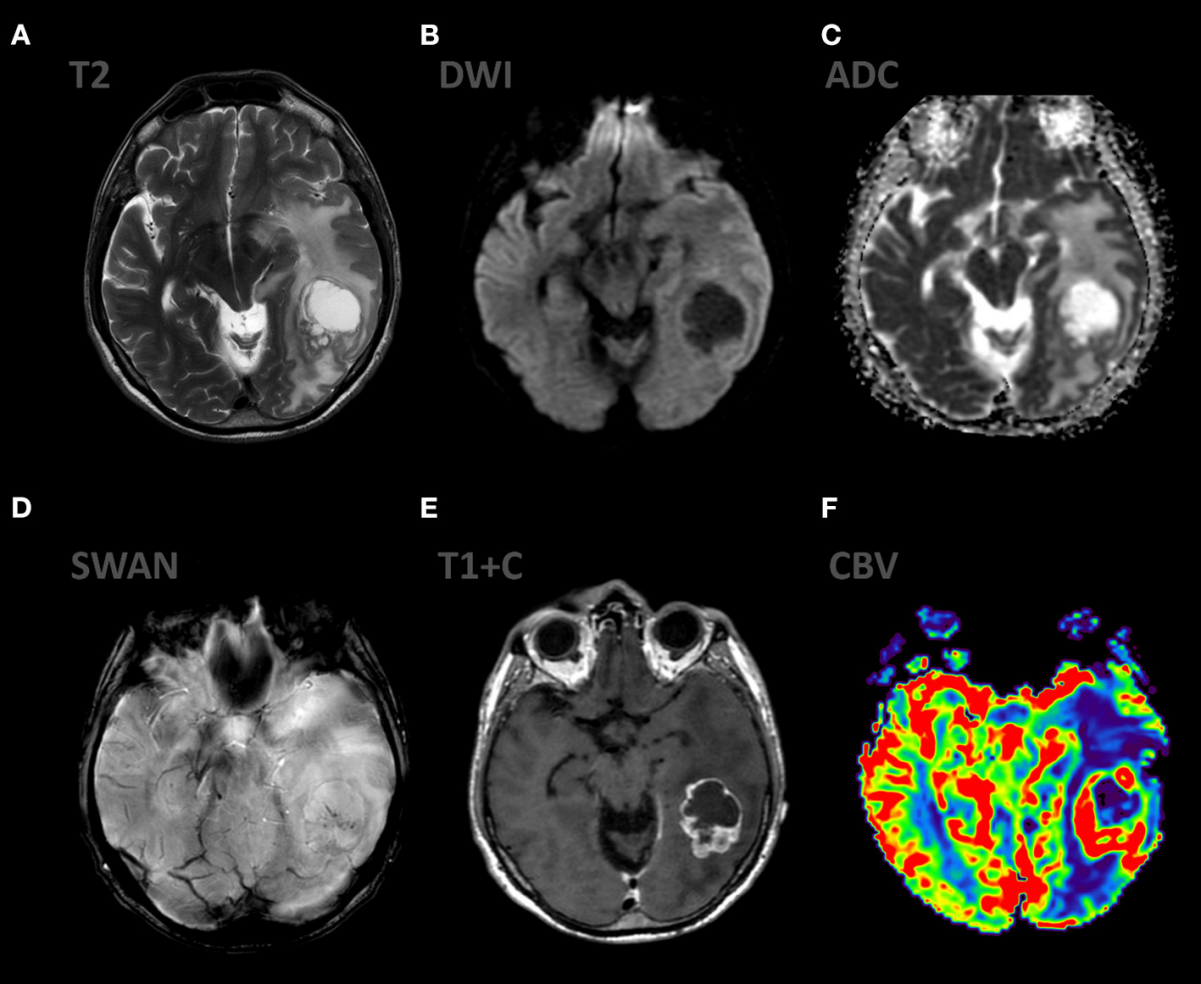
mpMRI ax: **(A)** T2WI; 6, **(B)** DWI, **(C)** ADC, **(D)** SWAN, **(E)** T1 + C, **(F)** DSC-PWI show classic appereance of solitary brain metastasis. There is a lesion with ring-like enhancing pattern and strong peripheral T2-hyperintensiry. Vasogenic edema is out of proportion with tumor size. The enhancing component of the lesion demonstrates high rCBV values.

Malignant gliomas are characterized by an annular contrast pattern with uneven wall thickness, reflecting necrosis (“crown effect”), a combination of vasogenic edema and a non-contrasting infiltrative tumor component, a distinct mass effect, and average values of the ADC in the range of 745 ± 135 × 10^−6^ mm^2^/s (for GBM), within 1,067 ± 276 × 10^−6^ mm^2^/s (for AA), elevation of rCBV values by 6.9 ± 3.12 times compared to unchanged white matter, the presence of intratumoral vascular shunts and a low-intensity rim due to the breakdown of products blood test for SWAN (1, 28–33) (Figure 11).

sMTS have radiological manifestations similar to those of GBM: a ring-shaped contrasting pattern with a central zone of necrosis, hemorrhage. The differences are the average values of the ADC within 919.4 ± 200 × 10^−6^ mm^2^/s, the absence of a diffuse tumor component in the vasogenic edema and the absence of a tendency to form pathological shunts, which explains the lower perfusion values (1, 28, 30, 32, 34).

In our studies, all cases of lymphomas corresponded to the classical radiological features (intense and homogeneous contrast enhancement, peritumoral vasogenic edema, and low intratumoral ADC values) except for 10 cases showing hypervascularization with rCBV values that exceeded the values of unchanged white matter by 3.0–7.5 times and hemorrhages that occurred in three cases (see Results), which, according to some authors, is extremely uncharacteristic for the lymphoproliferative process.

PCNS lymphomas may mimic high-grade glioma or metastatic disease, showing a similar morphology and infiltrative growth pattern. High grade gliomas and metastases are distinguished by the presence of neoangiogenesis, the biomarker of which is an increase in rCBV obtained using contrast MR (DSC-T2^*^) or perfusion CT. Our experience shows that the use of perfusion techniques, which are considered highly sensitive and highly specific for assessing neovascularization, does not always make it possible to distinguish lymphoma from other aggressive brain tumors, since, despite the absence of neoangiogenesis, an increase in rCBV values is often found in PCNS lymphomas, which must be due to some interaction of the tumor with the vasculature of the brain. Instead of forming new ones, PLCNS infiltrates existing vessels. Neoplastic cells proliferate angiocentrically, surround the vessels in a “perivascular cuff” type, and diffusely or in the form of a well-defined front invade the healthy brain parenchyma. In the predominant number of our cases, among PLCNS with hyperperfusion, an increase in rCBV values is observed in a ring-like manner at the periphery of the tumor, where tumor cells interact with the vessels of the unchanged parenchyma.

Analysis of the data obtained from the assessment of immunoreactivity using antibodies to VEGF, CD3, CD20, CD31, CD34 established the absence of signs of neoangiogenesis and the presence of very high values of the proliferative activity index (Ki-67 > 90%), indicating a pronounced biological aggression.

It is noteworthy that against the background of diffuse hemorrhages into the tumor, detected in several patients, there were additional changes in the diameter of capillaries and arterioles according to the type of “vasodilation” with increased wall permeability, which may be due to inflammatory activity, the phase of inflammation or the duration of the process. The data obtained suggest that hyperperfusion of lymphomas is not associated with the activation of neoangiogenesis, as in the cases of glioblastomas and metastases, but is most likely associated with hemodynamic changes in the characteristics of blood vessels. Moreover, increased expression of VEGF (vascular endothelial growth factor) in lymphoma is associated with greater survival (8). Despite a similar increase in rCBV values in our observations, it is not possible to assume the presence of an identical mechanism of vascularization. It is noteworthy that similar mechanisms of an increase in the diameter of blood vessels and an increase in rCBV values, leading to vasodilation, have been described in multiple sclerosis during the formation of a new acute lesion before the disintegration of the blood-brain barrier or during reactivation of chronic foci (35). Perfusion MR or CT data, which make it difficult to distinguish lymphoma from other aggressive intracerebral tumors, are probably due to the immune response of healthy vessels of the border zone to PLCNS cell invasion and perivascular infiltration (36–38).

## Conclusions

Today, if a neuroradiologist is faced with the task of making a differential diagnosis of intracerebral formations, performing multiparametric MRI mapping is a key condition for obtaining the necessary information about the nature of the tumor. It is generally accepted that knowledge of perfusion parameters (CBV) is critical. However, our study demonstrates the lack of reliability of MR and CT perfusion in cases of PCNS lymphomas. Even mpMRI does not always reliably distinguish lymphoma from other malignant brain tumors (high-grade gliomas and metastases). The use of stereotactic biopsy with histopathological matching remains the “gold” standard in the diagnosis of PCNS lymphomas.

## Data Availability Statement

The raw data supporting the conclusions of this article will be made available by the authors, without undue reservation.

## Ethics Statement

Written informed consent was obtained from the individual(s), and minor(s)' legal guardian/next of kin, for the publication of any potentially identifiable images or data included in this article.

## Author Contributions

RT, TT, and AS: conceptualization. VM, AP, and OB: data curation. OB, AS, TI, and YG: formal analysis. RT: investigation. OB: project administration. RT and TT: resources. OB, RT, and TI: software. AS and TT: supervision. OB, TI, and TT: validation. RT and OB: roles/writing—original draft. RT and YG: writing—review and editing. All authors contributed to the article and approved the submitted version.

## Conflict of Interest

The authors declare that the research was conducted in the absence of any commercial or financial relationships that could be construed as a potential conflict of interest.

## Publisher's Note

All claims expressed in this article are solely those of the authors and do not necessarily represent those of their affiliated organizations, or those of the publisher, the editors and the reviewers. Any product that may be evaluated in this article, or claim that may be made by its manufacturer, is not guaranteed or endorsed by the publisher.

## References

[1] Hartmann M Heiland S Harting I Tronnier V Sommer C Ludwig R. Distinguishing of primary cerebral lymphoma from high-grade glioma with perfusion-weighted magnetic resonance imaging. Neurosci Lett. (2003) 338:119–22. 10.1016/S0304-3940(02)01367-8 12566167

[2] Hakyemez B Erdogan C Bolca N Yildirim N Gokalp G Parlak M. Evaluation of different cerebral mass lesions by perfusion-weighed MR imaging. J Magn Reson Imaging. (2006) 24:817–24. 10.1002/jmri.20707 16958061

[3] Wong ET. Management of central nervous system lymphomas using monoclonal antibodies: challenges and opportunities. Clin Cancer Res. (2005) 11:7151s−57s. 10.1158/1078-0432.CCR-1004-0002 16203815

[4] Mohile NA Abrey LE. Primary central nervous system lymphoma. Semin Radiat Oncol. (2007) 17:223–29. 10.1016/j.semradonc.2007.02.008 17591569

[5] Haldorsen IS Krakenes J Krossnes BK Mella O Espeland A. CT and MR imaging features of primary central nervous system lymphoma in Norway, 1989–2003. AJNR Am J Neuroradiol. (2009) 30:744–51. 10.3174/ajnr.A1447 19164442 PMC7051753

[6] Sugahara T Korogi Y Shigematsu Y Hirai T Ikushima I Liang L. Perfusion-sensitive MRI of cerebral lymphomas: a preliminary report. J Comput Assist Tomogr. (1999) 23:232–7. 10.1097/00004728-199903000-00011 10096330

[7] Koeller KK Smirniotopoulos JG Jones RV. Primary central nervous system lymphoma: radiologic-pathologic correlation. Radiographics. (1997) 17:1497–526. 10.1148/radiographics.17.6.9397461 9397461

[8] Trofimova TN Savintseva ZHI Skvortsova TY. Monography: Cerebral Glial Tumors Radiology.(2020).

[9] Iwamoto FM Abrey LE. Primary dural lymphomas: a review. Neurosurg Focus. (2006) 21:E5. 10.3171/foc.2006.21.5.6 17134121

[10] Go JL Lee SC Kim PE. Imaging of primary central nervous system lymphoma. Neurosurg Focus. (2006) 21:E4. 10.3171/foc.2006.21.5.5 17134120

[11] Kuker W Nagele T Korfel A Heckl S Thiel E Bamberg M. Primary central nervous system lymphomas (PCNSL): MRI features at presentation in 100 patients. J Neurooncol. (2005) 72:169–77. 10.1007/s11060-004-3390-7 15925998

[12] Buhring U Herrlinger U Krings T Thiex R Weller M Kuker W. features of primary central nervous system lymphomas at presentation. Neurology. (2001) 57:393–96. 10.1212/WNL.57.3.393 11515505

[13] Senocak E Oguz KK Ozgen B Mut M Ayhan S Berker M. Parenchymal lymphoma of the brain on initial MR imaging: a comparative study between primary and secondary brain lymphoma. Eur J Radiol. (2010) 57:393–96. 10.1016/j.ejrad.2010.01.017 20202775

[14] Calli C Kitis O Yunten N Yurtseven T. Perfusion and diffusion MR imaging in enhancing malignant cerebral tumors. Eur J Radiol. (2006) 58:394–403. 10.1016/j.ejrad.2005.12.032 16527438

[15] Zacharia TT Law M Naidich TP Leeds NE. Central nervous system lymphoma characterization by diffusion-weighted imaging and MRspectroscopy. J Neuroimaging. (2008) 18:411.17. 10.1111/j.1552-6569.2007.00231.x 18494774

[16] Schroeder PC Post MJ Oschatz E Stadler A Bruce-Gregorios J Thurnher MM. Analysis of the utility of diffusionweighted MRI and apparent diffusion coefficient values in distinguishing central nervous system toxoplasmosis from lymphoma. Neuroradiology. (2006) 48:715–20. 10.1007/s00234-006-0123-y 16947010

[17] Schlegel U Schmidt-Wolf IG Deckert M. Primary CNS lymphoma: clinical presentation, pathological classification, molecular pathogenesis and treatment. J Neurol Sci. (2000) 181:1–12. 10.1016/S0022-510X(00)00385-3 11099705

[18] Eichler AF Batchelor TT. Primary central nervous system lymphoma: presentation, diagnosis and staging. Neurosurg Focus. (2006) 21:E15. 10.3171/foc.2006.21.5.16 17134117

[19] Coulon A Lafitte F Hoang-Xuan K Martin-Duverneuil N Mokhtari K Blustajn J. Radiographic findings in 37 cases of primary CNS lymphoma in immunocompetent patients. Eur Radiol. (2002) 12:329–40. 10.1007/s003300101037 11870430

[20] Thurnher MM Rieger A Kleibl-Popov C Settinek U Henk C Haberler C. Primary central nervous system lymphoma in AIDS: a wider spectrum of CT and MRI findings. Neuroradiology. (2001) 43:29–35. 10.1007/s002340000480 11214644

[21] Fine HA Mayer RJ. Primary central nervous system lymphoma. Ann Intern Med. (1993) 119:1093–104. 10.7326/0003-4819-119-11-199312010-00007 8239229

[22] Haldorsen IS Krakenes J Goplen AK Dunlop O Mella O Espeland A. AIDS-related primary central nervous system lymphoma: a Norwegian national survey 1989–2003. BMC Cancer. (2008) 8:225. 10.1186/1471-2407-8-225 18684320 PMC2525658

[23] Hill QA Owen RG. CNS prophylaxis in lymphoma: who to target and what therapy to use. Blood Rev. (2006) 20:319–32. 10.1016/j.blre.2006.02.001 16884838

[24] Bierman P Giglio P. Diagnosis and treatment of central nervous system involvement in non-Hodgkin's lymphoma. Hematol Oncol Clin North Am. (2005) 19:597–609. 10.1016/j.hoc.2005.05.003 16083825

[25] Wu O Ostergaard L Sorensen AG. Technical aspects of perfusion-weighted imaging. Neuroimaging Clin N Am. (2005) 15:623–37, xi. 10.1016/j.nic.2005.08.009 16360593

[26] Cianfoni A Colosimo C Basile M Wintermark M Bonomo L. Brain perfusion CT: principles, technique and clinical applications. Radiol Med. (2007) 112:1225–43. 10.1007/s11547-007-0219-4 18074193

[27] Fainardi E Di BF Borrelli M Saletti A Cavallo M Sarubbo S. Potential role of CT perfusion parameters in the identification of solitary intra-axial brain tumor grading. Acta Neurochir Suppl. (2010) 106:283–87. 10.1007/978-3-211-98811-4_53 19812965

[28] Lee IH Kim ST Kim HJ Kim KH Jeon P Byun HS. Analysis of perfusion weighted image of CNS lymphoma. Eur J Radiol. (2009). 10.1016/j.ejrad.2009.05.013 19500931

[29] Kremer S Grand S Remy C Esteve F Lefournier V Pasquire B. Cerebral blood volume mapping by MR imaging in the initial evaluation of brain tumors. J Neuroradiol. (2002) 29:105–13. 12297732

[30] Savintseva ZHI Skvortsova TY Trofimova TN Gurchin AF Smirnov AV. Differentiation between brain tumor recurrence and radiation injury using diffusion-weighted imaging and perfusion magnetic resonance imaging. Folia. Neuropathol. (2015) 48:81–92. 10.22328/2079-5343-2015-4-27-34 20602289

[31] Law M Yang S Babb JS Knopp EA Golfinos JG Zagzag D. Comparison of cerebral blood volume and vascular permeability from dynamic susceptibility contrast-enhanced perfusion MR imaging with glioma grade. Am J Neuroradiol. (2004) 25:746–55. 15140713 PMC7974484

[32] Rollin N Guyotat J Streichenberger N Honnorat J Tran Minh VA Cotton F. Clinical relevance of diffusion and perfusion magnetic resonance imaging in assessing intra-axial brain tumors. Neuroradiology. (2006) 48:150–9. 10.1007/s00234-005-0030-7 16470375

[33] Lemercier P Paz Maya S Patrie JT Flors L Leiva-Salinas C. Gradient of apparent diffusion coefficient values in peritumoral edema helps in differentiation of glioblastoma from solitary metastatic lesions. AJR Am J Roentgenol. (2014 J) 203:163–9. 10.2214/AJR.13.11186 24951211

[34] Zakaria R Das K Radon M Bhojak M Rudland PR Sluming V Jenkinson MD. Diffusion-weighted MRI characteristics of the cerebral metastasis to brain boundary predicts patient outcomes. BMC Med Imaging. (2014) 3:14–26. 10.1186/1471-2342-14-26 25086595 PMC4126355

[35] Wuerfel J Bellmann-Strobl J Brunecker P Aktas O McFarland H Villringer A. Changes in cerebral perfusion precede plaque formation in multiple sclerosis: a longitudinal perfusion MRI study. Brain. (2004), 127:111 ± 119. 10.1093/brain/awh007 14570816

[36] Blasel S Vorwerk R Kiyose M Mittelbronn M Brunnberg U Ackermann H. New MR perfusion features in primary central nervous system lymphomas: pattern and prognostic impact. J Neurol. (2018) 265:647–58. 10.1007/s00415-018-8737-7 29383512

[37] Sugita Y Takase Y Mori D Tokunaga O Nakashima A Shigemori M. Endoglin (CD 105) is expressed on endothelial cells in the primary central nervous system lymphomas and correlates with survival. J Neurooncol. (2007) 82:249–56. 10.1007/s11060-006-9281-3 17102906

[38] Takeuchi H1 Matsuda K Kitai R Sato K Kubota T. Angiogenesis in primary central nervous system lymphoma (PCNSL). J Neurooncol. (2007) 84:141–5. 10.1007/s11060-007-9363-x 17406788

